# The Bug under the Chip

**Published:** 1891-07

**Authors:** 


### THE "BUG UNDER THE CHIP."

"The Association is losing its best and most useful members—what is to be done?" whines a certain "recent" member, in great solicitude.

When the Secretary read two resignations at Waco a certain saintly member rose to inquire if any *reason* was assigned; and would have been glad to make somebody believe that it was on account of the Secretary. No reason was assigned; but shortly after the meeting the JOURNAL received through the mail an advertisement cut from the *Houston Post*,—six by four inches,—in which the name of one of the resigners appeared in block letters an inch high—followed by the claim that he had the only genuine Koch's lymph in Houston, and that he was curing consumption right straight along—five aud ten cases a day "hand-running;" that he was a "specialist" from away back—(none genuine without the trade mark)—that cross eyes were cured in a minute,—likewise catarrh; and calling on everybody to come to his office, and if they didn't believe it he would show them the lymph, and the scissors and the "cross" taken out of some eyes that very day; in fact, it was a *model* quack advertisement; and *this* was perhaps the reason why *one* of the "best and most useful members" was "lost to the Association."—See?

